# Functional Deficits and Diffusion Tensor Magnetic Resonance Imaging of the Olfactory and Vision Systems in the Alzheimer’s Disease Model of Aging GluN3A Knockout Mice †

**DOI:** 10.3390/cells15211951

**Published:** 2026-10-27

**Authors:** Ling Wei, Michael Qize Jiang, Yingying Zhao, Daiana Takashima, Yi Li, Khan Hekmatyar, Lingyue Wu, Ananth Sastry, Jenny J. Yang, Shan Ping Yu

**Affiliations:** 1 Department of Anesthesiology, Emory University School of Medicine, Atlanta, GA 30322, USA; lwei7@emory.edu (L.W.); michealjiang@emory.edu (M.Q.J.); zyytx@sina.com (Y.Z.); daiana.takashima@emory.edu (D.T.); belindaliyi_123@126.com (Y.L.); lingyue.wu@emory.edu (L.W.); ananth.sastry@emory.edu (A.S.); 2 Department of Neurology, Beijing Friendship Hospital, Beijing 100052, China; 3 Department of Neurology, Beijing Hospital, National Center of Gerontology, Institute of Geriatric Medicine, Chinese Academy of Medical Sciences, Beijing 100730, China; 4 Department of Chemistry, Advanced Translational Imaging Facility (ATIF), Georgia State University, Atlanta, GA 30303, USA; nhekmatyar@gsu.edu (K.H.); jenny@gsu.edu (J.J.Y.); 5 Center for Visual and Neurocognitive Rehabilitation, Atlanta Veterans Affairs Medical Center, Decatur, GA 30033, USA

**Keywords:** GluN3A deficiency, sporadic AD, neural network connection, white matter damage, brain atrophy, degenerative excitotoxicity, DTI, MRI

## Abstract

Sensory dysfunction, such as olfactory and visual deficits, is a notorious symptom during the progression of Alzheimer’s disease (AD) and related dementia (ADRD). The causal factor/s and structural disruptions underlying the sensory dysfunction in the AD/ADRD brain are not well understood. The NMDA receptor subunit GluN3A (NR3A) negatively regulates the receptor activity to uphold cellular Ca^2+^ homeostasis. In GluN3A knockout (KO) mice, animals spontaneously develop chronic neurodegenerative excitotoxicity, cognitive decline, and AD-like pathology. Using diffusion tensor imaging (DTI), we examined neuronal network destruction together with sensory dysfunction in the GluN3A KO mouse. Specific attention was given to the selective vulnerability of the olfactory and visual nuclei, white matter injury in regional connections between subcortical centers and the cortex and hippocampus. Compared to age-matched wild-type (WT) controls (male), consistent with our early reports in younger ages, 18-month-old aging/aged GluN3A KO mice (male) suffered significant olfactory, visual and social deficits, accompanied by marked Aβ42 deposition in the brain. Ex vivo MR images of the GluN3A KO and control brains were acquired on the 7T MRI system, using a four-channel mouse array RF coil. High-resolution 3D T2-weighted RARE anatomical and DTI scans were performed. The DTI analysis revealed that GluN3A KO mice developed brain atrophy, involving significant damage in the white and gray matter of the olfactory and visual systems and impaired pathways among the superior colliculus, lateral geniculate nucleus, and the hippocampus and/or the cortex. This investigation disclosed a receptor-mediated neurodegeneration that explains sensory syndromes featured in a sporadic AD mouse model.

## 1. Introduction

Alzheimer’s disease (AD) is characterized by age-dependent progressive dementia accompanied by the generation of amyloid β (Aβ) plaques and neuronal fibrillary tangles (NFTs) [1,2]. Synaptic disruption and neuronal loss in brain regions critical for cognition, such as the neocortex and hippocampus, are structural features of AD [1,3]. The amyloid hypothesis proposes an initiating event of amyloidosis and/or increased Aβ oligomers, with tau protein accumulation, causing neurodegeneration and cognitive decline. The Aβ and tau pathology is assumed responsible for both familial AD (FAD) and sporadic AD (SAD) [1,2]. However, increasing evidence indicates SAD pathogenesis is more complex and follows a distinctive prolonged time course (years to decades) of progression before clinical diagnosis [4,5]. Clinical observations indicate that the risk and functional deficits of AD can diverge from the extent of amyloid deposition [6]. In >95% of cases of late-onset SAD, pathophysiological cascades progress prior to significant amyloid deposition, cognitive symptoms, and clinical diagnosis, while several early symptoms emerge in the preclinical stage of the disease [7]. Common signs of initial and lasting neurodegeneration are often associated with deteriorations of the sensory systems, leading to functional deficits in the visual, olfactory, and/or auditory abilities [7]. The precise initial trigger for these earliest symptoms and associated neurodegeneration has been obscure and often overlooked in AD research. Increasing basic and clinical evidence suggests that both amyloid-dependent and -independent mechanisms should be considered [8]. A better understanding of the pathogenesis and pathology focusing on SAD appears necessary for developing effective therapies for most common SAD patients.

Olfactory dysfunction is a general sign of neurodegeneration before mild cognitive impairment (MCI) and AD [9]. Approximately 90% of AD and Parkinson’s disease (PD) patients experience deterioration of olfactory sensation, while AD patients are affected more than PD patients [10,11]. The olfactory epithelium (OE) and olfactory bulb (OB) receive the sensation of smell and send the sensory input to the entorhinal cortex, which projects to the hippocampus to form olfactory learning/memory outputs [12]. In animal studies, OB denervation causes hippocampus-dependent memory loss, whereas, interestingly, OB stimulation promotes cognitive function [13,14]. As a hallmark of AD, Aβ deposition appears early in sensory association areas [15,16]. NFTs exist in the OB, olfactory tracts, the trans-entorhinal and entorhinal cortex, and the anterior olfactory nucleus [17]; their numbers are correlated with the severity of dementia. Thus, amyloid peptides and tau pathology contribute to sensory deterioration [11,18,19,20]. On the other hand, the structural impairments and root triggers of sensory network damage have not been explicitly delineated in AD animal models or clinical cases.

Vision deficits are commonly observed in patients with AD and ADRD, causing problems in color perception, contrast sensitivity, depth perception, and object recognition [21,22,23].

Previous studies demonstrated that various types of vision problems are linked to an increased risk of cognitive impairment [24]. In particular, posterior cortical atrophy (PCA) is a visual variant of AD, while glaucoma, age-related macular degeneration, cataracts, and diabetes-related eye disorders are associated with increased risk for dementia [24]. The deficits can result from structural changes in the retina and/or the brain’s visual processing area, such as the occipital and parietal lobes. Unlike visual symptoms caused by problems in the eye itself, the AD-related visual impairment stems from the inability to transport and/or interpret visual signals from the eyes [23]. The lateral geniculate nucleus (LGN) is a midbrain structure important for visual–motor transformations, particularly eye movements. Pathways involving the superior colliculus (SC) are crucial for orienting the body toward stimuli and include connections from the retina and visual cortex, as well as projections to the brainstem and other motor areas. In normal visual function, the vast majority of optic tract fibers terminate on neurons in the subcortical visual center of the LGN in the thalamus [25]. The optic tract fibers from each eye form synapses in different layers of the LGN, which is critical for relaying signals from the retina to the primary visual cortex [26]. Structural alterations of visual pathways in AD have drawn attention in the last few years; the focus, however, has been primarily on glaucoma and associated disorders [27]. It was proposed that visual circuit disturbances in both the early and late stages of AD provide anatomical and functional insights into the visual circuitry of AD [28].

The N-methyl-D-aspartate receptor (NMDAR) plays a pivotal role in neurophysiology and neuropathology [29,30]. Neuronal and NMDAR hyperactivity is a shared characteristic of AD in humans and animal models, directly contributing to synaptic dysfunction by mediating Ca^2+^ influx into excitatory neurons [29,30]. In GluN3A knockout (KO) mice, a genetic deficit of the NMDAR regulatory subunit GluN3A (NR3A), we recently identified GluN3A deficiency as a novel pathogenic factor in late-onset SAD [31]. As in human cases, mice do not exhibit a disease phenotype in young adulthood, although neuronal hyperactivity and mild yet persistent [Ca^2+^]_i_ increases exist in the brain [31]. Sensory deficits, such as decreased olfactory sensation and psychological alterations, emerge as early syndromes followed by cognitive decline. GluN3A KO mice spontaneously undergo endogenous amyloid/tau pathology in a much delayed manner, i.e., after, but not before, cognitive decline [31]. In a modified Ca^2+^ hypothesis, we proposed that, independent of amyloid toxicity, mild but sustained increases in [Ca^2+^]_i_ can trigger specific molecular and cellular events leading to delayed cell death, chronic inflammation, and pathological changes, such as synapse loss, neurovascular damage, mitochondrial dysfunction, and impaired neural networks [31]. More evidence suggests that the Ca^2+^ increase may be linked directly and indirectly to presenilin mutations, ApoE4 expression, synaptic dysfunction, and Aβ/tau pathology [32].

Although our investigation identified typical AD pathology and functional deficits, including the early syndrome of sensory and sensorimotor dysfunction of GluN3A KO mice [31,33], the structural mechanism underlying the abnormal sensory capability is obscure in this and other AD models. The present investigation is intended to examine disruptions of neuronal networks and axonal connections important for olfactory and visual functions in the SAD mouse brain.

Advances in MRI technologies have enabled the detection of several phenotypes relevant to AD, including brain atrophy, alterations in the connectome, and ultrastructural organization [34]. Importantly, Diffusion Tensor Imaging (DTI) can detect changes in white matter and gray matter, showing how different brain regions connect, and how these changes manifest as behavioral phenotypes [35]. We hypothesize that analyzing differences at the network level of brain structures would be sensitive to detecting differences in higher brain functions. This approach enables us to relate pathological behavioral phenotypes to neurodegenerative changes in the brain [36]. The information may shed light not only on neuropathology but also help identify therapeutic targets for the treatment of AD and ADRD.

## 2. Methods and Materials

### 2.1. Ad Model of the Glun3a Ko Mouse

GluN3A KO mice and wild-type (WT) counterparts were initially provided by Nobuki Nakanishi and Stuart A. Lipton at Sanford-Burnham Medical Research Institute (La Jolla, CA, USA). Detailed information on the background of these mice was described previously [37]. Briefly, embryonic stem cells derived from 129/SvJ were electroporated with DNA carrying a disrupted GluN3A gene and then injected into blastocysts from C57BL/6 mice. The resulting chimeric males were crossed with black Swiss or 129SvEv females to produce F1 heterozygotes.

GluN3A KO homozygous mice were then produced by crossbreeding F1 mice. Homozygous colonies of WT or GluN3A KO mice were maintained under identical animal care environments. Mice were housed in standard rodent cages in the Emory University animal facility, where the room temperature was kept at 22 ± 1 °C, and light was controlled at a 12 h light/12 h dark daily cycle. Aging (~18-month-old) male littermate WT and KO animals were tested in the experiments. DNA genotyping was performed to confirm the animals’ phenotypes [33].

The study was conducted according to the guidelines of the NIH and Emory’s institutional policy on the research use of rodent animals. The animal use was reviewed and approved by the Emory University Institutional Animal Care and Use Committee (IACUC: PROTO201701059, 10 August 2022).

### 2.2. Dti-Mri

High-resolution ex vivo MR imaging of isolated brains was acquired on the 7T MRI system (Bruker Biospec, Billerica, MA, USA), with a four-channel mouse radiofrequency array coil. The brains were prepared in and removed from the 4% PFA solution, patted dry, and transferred to a 10 mL tube containing the Gd:PBS (1:200) solution for 24 h before being placed in a Fomblin solution, which is a proton-free fluid used in ex vivo MRI to reduce magnetic susceptibility artifacts. The solution produces no MRI signal and has a magnetic susceptibility similar to that of tissue. The brain was then placed in a 3D-printed mold and inserted into a 15 mL tube containing the Fomblin solution; the entire tube was inserted into the coil. After performing a localizer scan and optimizing the magnetic field homogeneity using the PRESS sequence and B0 map, a high-resolution 3D T2-weighted RARE anatomical scan was collected with the following parameters: TR/TE = 800/30 ms; matrix size = 106 × 100 × 113 with isotropic resolution of 150 μm. The total acquisition time for this scan was 20 min. Next, a high-resolution spin echo 3D DTI scan was collected with the following parameters: TR/TE = 1000/27.4 ms; four EPI segments; bandwidth of 300 kHz; and 65 non-collinear gradient directions with a single b-value shell at 4000 s/mm^2^ and five images with a b-value of 0 s/mm^2^ with the same resolution as the 3D anatomical scan. The total scan time for DTI was approximately 2 h for each animal. Once collected, all DTI scans were converted to NIFTI files and processed via Diffusion Spectrum Imaging (DSI) studio.

Diffusion MR images were processed using DSI (available at https://dsi-studio.labsolver.org/download.html) (accessed on 1 May 2024–30 October 2025). The brain was first masked, and motion correction was performed. Reconstruction of the DTI tractography was achieved by performing an Eigenvector analysis on the calculated tensor. Tractography was generated using a tracking plan (Trilinear interpolation in all orientations) which terminated after generating 2 million fiber tracts with lengths between 0.5 and 300 mm, utilizing all fiber directions at a turning angle of 45 degrees, with smoothing and a step size of 0.05 mm with a threshold fractional anisotropy (FA) of 0.02. The total number of streamlines of the whole brain and individual ROIs were recorded.

### 2.3. The Olfactory Habituation/Dishabituation Test

The olfactory habituation/dishabituation test was performed for odor detection. We placed the cotton-tipped testing stick containing water or a specific odor on the cage top, 2–3 cm from the mouse. Mice were initially presented with water for 2 min and repeated 3 times. The object was then given a novel vanilla-containing bar for 2 min and repeated 3 times. Lastly, a novel almond was presented for 2 min and repeated 3 times. The animal sniffing time directed toward the odor was recorded and off-line analyzed.

### 2.4. The Visual Platform Detection Test

This is a practical test designed for the visual response of mice [38]. The mouse is lifted by grasping its tail base and gradually lowered onto the flat surface of a table. While visually perceiving the approach of a surface, normal mice naturally extend their forepaws forward as an attempt to grasp. The distance at which the mouse starts the paw extension is measured to assess the visual placing response.

### 2.5. Three-Chamber Social Activity Test

The three-chamber sociability and social novelty tests were used to study social affiliation and social memory involving rodent sensory function. Mice were initially allowed to explore the three-chamber box for 10 min. The social behavior test was then performed by introducing a random littermate mouse into one of the side chambers for 10 min. The social novelty test was then performed by introducing a new stranger mouse into the chamber and moving the familiar littermate mouse to the other side. The time the testing mouse spent sniffing different animals was analyzed.

### 2.6. Barnes Maze Test

The Barnes maze test is similar to the widely used Morris water test for assessing learning and working memory, but without the stressful pressure of forced swimming [39]. Mice underwent a four-day training protocol using a modified Barnes maze paradigm. On Day 1, a habituation phase was conducted. Animals were first placed in the escape cage for 2 min, followed by a 30 s exposure to a clear beaker in the center of the maze. For maze exploration, animals were guided to the target hole for 15–30 s, then allowed up to 3 min to find and enter the escape hole. If the animal failed to enter the escape hole within 3 min, a gentle nudge was given at the 3-min mark. This was followed by two training trials (Trial 1 and Trial 2), each of which was preceded by a 15-s exposure to an opaque beaker in the center of the maze. In each trial, animals were allowed up to 3 min to explore the maze and locate the escape hole, followed by 1 min in the escape cage. A rest period of at least 30 min was provided between trials. On Days 1–3, three additional training trials were conducted and analyzed. Retention memory was tested 1 day after the training protocol; the time the mouse took to locate the escape hole and the travel time within the target area were recorded using the TopScan Realtime Option Version 3.0 (Clever Sys Inc., Reston, VA, USA) and compared between groups.

### 2.7. Statistical Analysis

All the data analysis, including image analysis and axon/cell counting, was performed in a blind or double-blind design; the researchers performing the test had no information about the genotype, age, or treatment. GraphPad Prism 9 (GraphPad Software, San Diego, CA, USA) was used for statistical analysis and graphic presentation. Unpaired Student’s *t*-test or Chi-squared test was used for pairwise comparisons. One-way or two-way ANOVA followed by Bonferroni or Fisher’s post-hoc test was used for multi-group comparisons. Analysis of multiple measurements acquired in the same animal over time was performed using a repeated-measures two-way ANOVA with linear mixed models, with fixed effects for genotype and time and random effects for animals. The group difference was considered significant when the *p* value was less than 0.05. Data are presented as Mean ± SEM.

## 3. Results

### 3.1. Ad Phenotype Evaluation of the Aging Glun3a Ko Mouse

**Figure 1:**
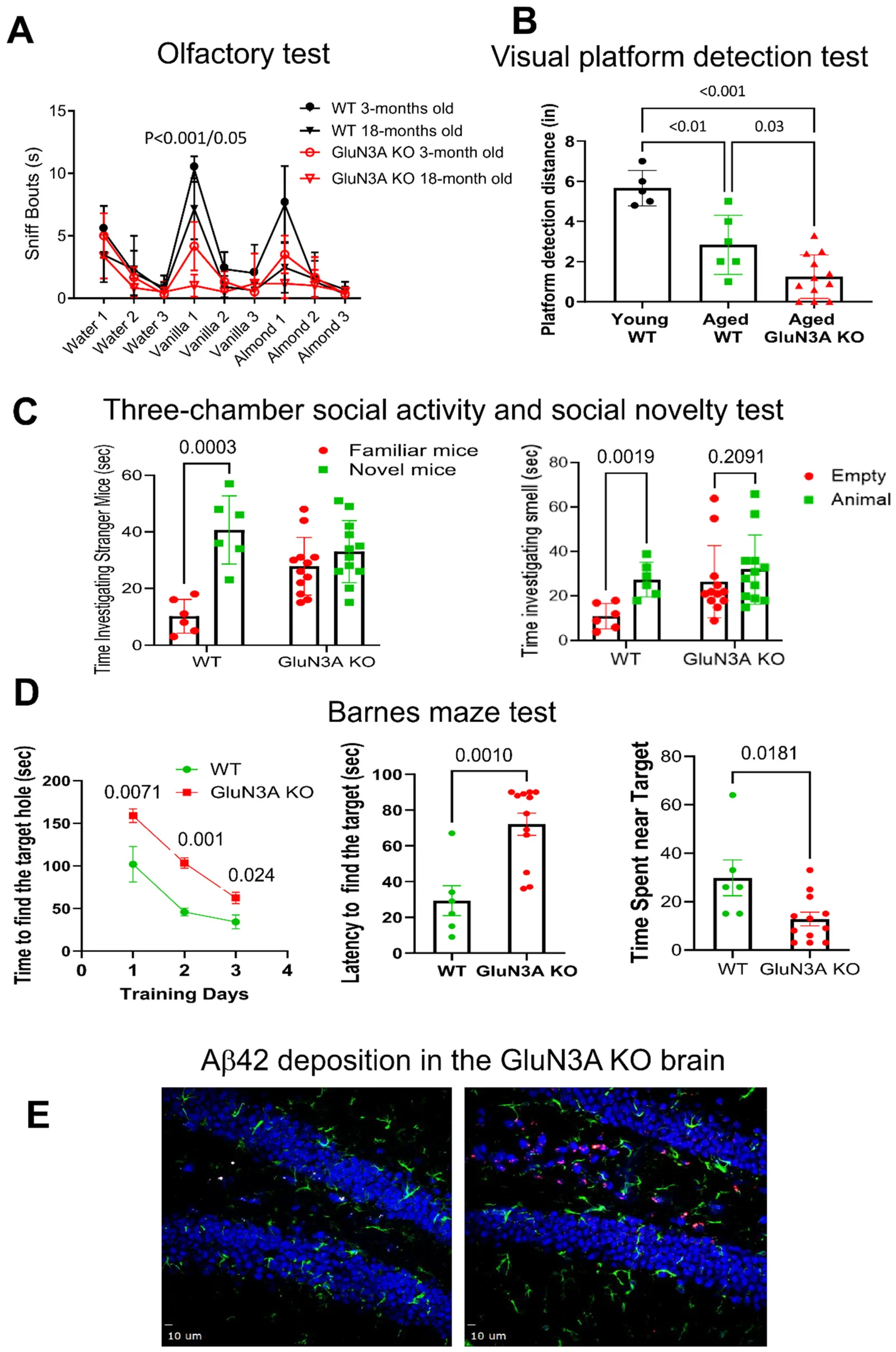
Behavioral and functional phenotypes of aging GluN3A KO mice. Social, olfactory, and visual functions were assessed using multiple tests in GluN3A KO mice and age-matched WT controls. (**A**). The olfactory habituation/dishabituation test reveals how animals respond to novel and familiar odors. There was no significant difference among all groups in the response to water. Significantly different responses were detected with the first application of the vanilla stimulus; the strongest sniffer response was observed in young adult (3-month-old) WT mice (served as a normal control in this test). Aged WT mice of 18 months old had a reduced response to vanilla (*p* = 0.004 vs. 3-month-old WT). At the young age of 3 months, GluN3A KO mice already displayed a reduced response to vanilla (*p* < 0.001 vs. 3-month-old WT), as an early sign of neurodegeneration in AD development. At 18 months of age, the olfactory deficit of GluN3A KO mice further deteriorated (*p* = 0.0039 vs. 3-month-old KO mice). In addition, all animals exhibited much reduced response to the s and third vanilla applications, which were no longer a novel stimulus to them. In response to the next novel odor stimulus of almond, young WT mice showed a renewed interest in the first almond, while aged WT mice and GluN3A KO mice had significantly reduced responses compared to young WT mice (*p* < 0.001). Two-way ANOVA with Tukey’s correction. N = 8/group. (**B**). In the visual platform detection test, compared to young adult animals 3 months of age, both aged WT and KO animals had significantly reduced visual function, detecting the platform only at a significantly shorter distance (*p* = 0.001 and *p* < 0001, respectively). Compared to WT mice, aged GluN3A KO mice performed even worse in perception of the table surface (*p* < 0.0001 and *p* < 0.05 vs. young and aged WT mice, respectively). Impressively, three mice in the aged GluN3A KO group completely failed to visually detect the platform. Two-way ANOVA with Tukey’s correction. N = 5 for young WT, n = 6 for aged WT, and n = 12 for aged GluN3A KO group. (**C**). The three-chamber social activity and social novelty test examined social behavior in 18-month-old WT and GluN3A mice. WT mice exhibited a marked interest in sniffing the novel partner, while this increased behavior was not observed in GluN3A KO mice. One-way ANOVA with Tukey’s correction; N = 6 for WT, n = 12 for GluN3A KO group. (**D**). The Barnes maze test was performed to test spatial learning and memory function of aged animals. This test, like the water maze test, includes a training phase for learning capability followed by a probe test of memory. During the 4 training days, 18-month-old GluN3A KO mice were much slower to identify the escape hole on the platform, as evidenced by prolonged delays each day of training compared to WT controls. In the following probe trial, GluN3A KO mice took much longer to find the target hole and spent less time in the targeted area. N = 6 for WT, n = 12 for GluN3A KO group; two-way ANOVA for the training curve, one-way ANOVA for latency and time around the target area. (**E**). Immunohistochemical staining of Aβ42 in the hippocampus of WT and GluN3A KO brains. Significant Aβ42 deposition (red) emerged in the 12-month-old GluN3A KO brain; no similar deposition was seen in the normally aging brain. The A deposition overlaid with the astrocyte marker GFAP (green) or the neuronal marker NeuN (blue).

Although we previously revealed AD development in young adult and adult (3–12 months old) GluN3A KO mice, little information was available on aging/aged GluN3A KO mice. To verify the AD pathology and behavioral changes, some key AD-associated deficits were examined in older animals. First, we inspected the olfactory and visual function of 18-month-old GluN3A KO mice and age-matched WT controls. The olfactory habituation/dishabituation test detected that aging GluN3A KO mice developed a severe deficiency in responding to a novel odor stimulus (Figure 1A). In the visual platform detection test, compared to young adult animals of 3 months old, both aged WT and KO animals suffered diminished capability in perceiving the platform (Figure 1B). Between these two groups, the GluN3A KO mouse performed even worse than normal aging WT controls (Figure 1B). More evidently, some GluN3A KO animals completely failed to visually detect the platform in this test (Figure 1B).

The three-chamber social activity test was applied to assess social recognition and social activity of aging GluN3A KO and WT mice. WT animals displayed a clear preference for spending time sniffing a strange mouse versus sniffing a familiar one (Figure 1C). In contrast, GluN3A KO mice showed no apparent preference for interacting with the stranger (Figure 1C), indicating poor social activity; meanwhile, reduced sensory functions might be a contributor factor.

To validate a more specific AD functional phenotype of the aging GluN3A KO mouse, the Barnes maze test was performed to examine spatial learning and memory [38]. During the training phase, GluN3A KO mice exhibited a significant shortcoming in learning, as evidenced by much longer escape latencies on training days 1, 2, and 3 compared to WT mice (Figure 1D). In the probe trial after training, GluN3A KO mice had significantly longer delay in locating the escape hole (Figure 1D) and spent less time inside the target quadrant compared to WT controls (Figure 1D). The results confirmed that these aging GluN3A KO mice suffered from a substantial disability in spatial learning and associated memory.

Confirming the landmark AD pathology of GluN3A KO mice, our previous studies detected Aβ plaque deposition and tau hyperphosphorylation spontaneously emerged in the GluN3A KO brain around 10–12 months of age [31]. In the current investigation, immunohistochemical staining showed abundant Aβ42 expression in the hippocampus (Figure 1E). The cognitive decline and massive Aβ deposition in the brain progressed without transgenic expression of exogenous AD genes, suggesting sporadic AD development in GluN3A KO mice.

### 3.2. Global Neurodegeneration of the Aging Glun3a Ko Mouse

**Figure 2:**
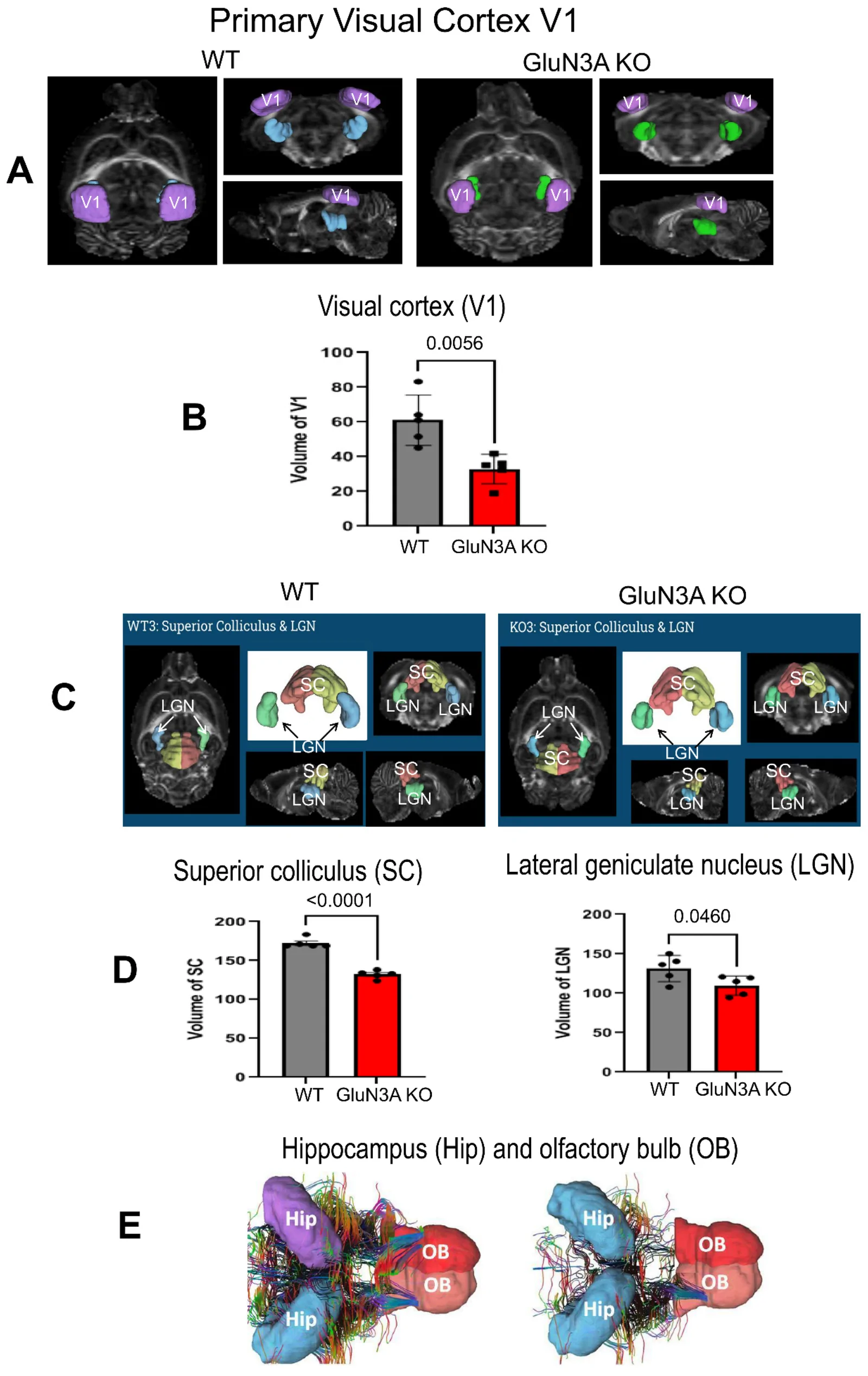
Brain atrophy and disrupted neuronal connections. The bilateral tractography of DTI was taken on the dissected brains of 18-month-old WT and GluN3A KO mice. (**A**). DTI images of brains showing the primary visual cortex (V1) region labeled in purple color, front and side of the brain. Green and blue colors show the lateral geniculate nucleus (LGN). (**B**). Volumetric analysis revealed a significant volume decrease in the visual cortex. *p* < 0.05. Unpaired *t*-test; n = 4 for the WT group and GluN3A KO group, respectively. (**C**,**D**). DTI images showing the superior colliculus (SC, red (right)/yellow (left)) and LGN (blue and green). (**C**). These subcortical visual processing structures underwent significant atrophy in the GluN3A KO brain, showing significant volume decreases (**D**). Unpaired *t* test; n = 4 in WT and GluN3A KO groups for the SC analysis, and n = 5 in each group for the LGN analysis. (**E**). A representative image showing the olfactory bulb (OB), the hippocampus (Hip) and connecting axonal tracts in WT and GluN3A KO brains. Color was generated randomly by the image software. Quantitative analyses of the volume and track number changes are reported in Table 1.

To investigate whether the GluN3A deficiency in the brain leads to broad disruptions of neuronal networks, we assessed the whole-brain tractography, specific brain regional volume, and neuronal connections of 18-month-old GluN3A KO mice and age-matched WT controls (Figure 2). Ex vivo diffusion tensor imaging (DTI) and volumetric analysis detected significant volume decreases of the olfactory bulb (OB) and the visual cortex (Table 1 and Figure 2A,B).

There were also trends of volume decrease of the hippocampus (*p* = 0.0733) and reduced connective tracts between the OB and hippocampus (*p* = 0.0955) in the GluN3A KO brain (Figure 2E and Table 1). Additional atrophy was detected in the SC and LGN nucleus (Figure 2C,D), which are important subcortical pathways to the visual cortex (V1), brainstem and thalamic areas [40].

### 3.3. Structural Disruptions of the Visual and Olfactory Systems in the GluN3A KO Brain

**Figure 3:**
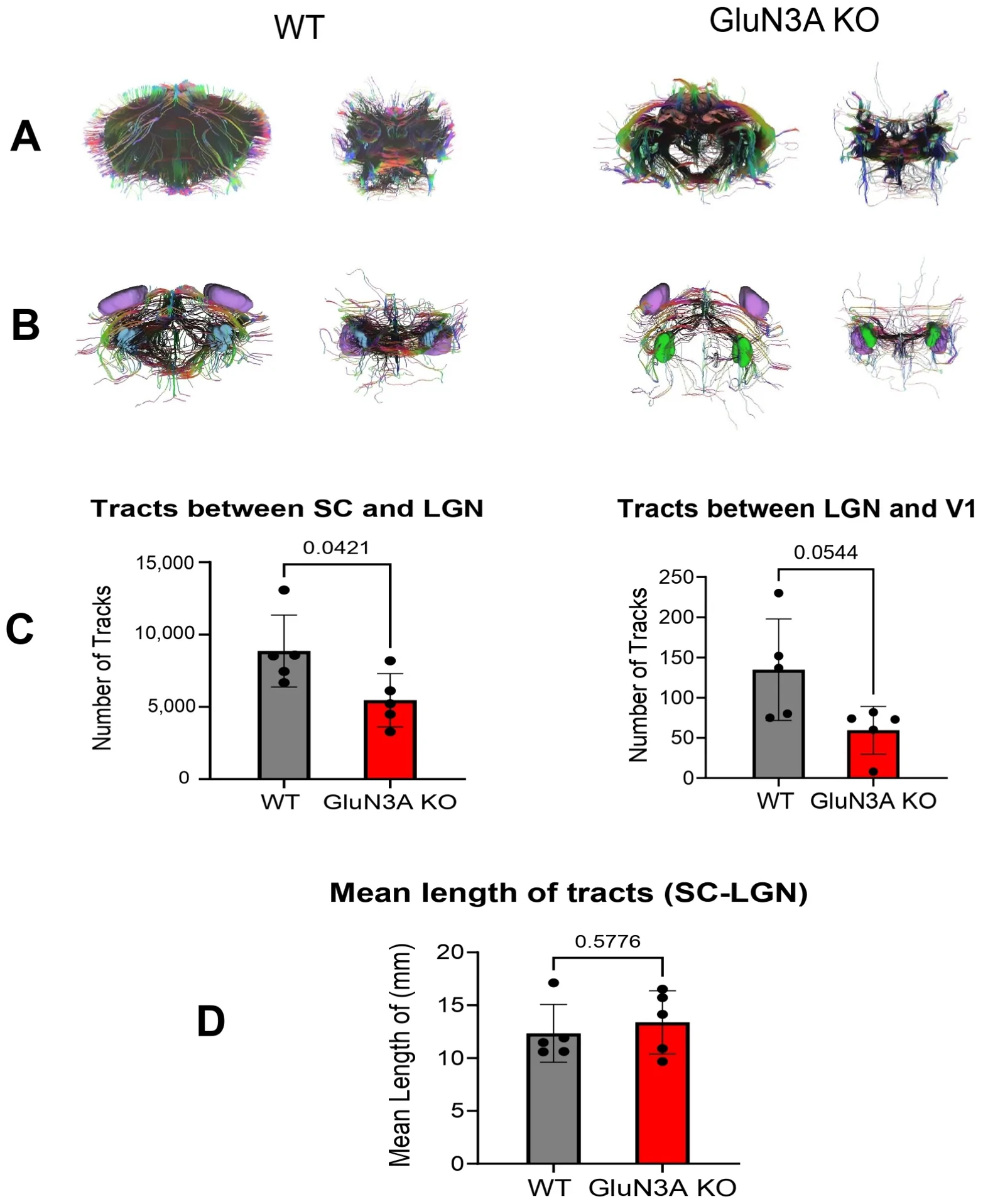
White matter degeneration and impaired axonal connections in the visual system: 3-D reconstructions of the tractography between the lateral geniculate nucleus and superior colliculus in GluN3A KO and WT brains. (**A**). The whole-brain tractography at the top and bottom levels of different mice. Axonal tracts are labeled with red or green colors; other colors were generated randomly by the imaging software. (**B**). Representative axonal tracts between the LGN are shown in blue and green in WT and GluN3A KO brains, respectively. The color purple indicates the V1 region. (**C**). Quantified analysis of the tract numbers between SC and LGN. There were significantly fewer tracts between the SC and LGN (**left bar graph**) and between the LGN and V1 (**right bar graph**) in the GluN3A KO brain compared to WT controls. (**D**). The mean length of tracts between SC and LGN was not significantly different between GluN3A KO and WT animals.

In addition to the gray matter atrophy, disruptions of the white matter of GluN3A KO mice highlighted a global impact of GluN3A deficiency on brain integrity and regional connectivity. Of clinical significance, the following studies examined the specific structural alterations of the olfactory and visual systems, respectively. DTI examinations on the specific axonal tracts disclosed significant reductions in the number of nerve tracts between SC and LGN and between LGN and the visual cortex V1 region (Figure 3C,D). The average length of axonal tracts between SC and LGN was not significantly different between WT and GluN3A KO mice (Figure 3D).

The above overall findings suggest that GluN3A KO mice exhibit significant volume loss in key brain regions involved in vision, olfaction, and cognitive processing, and that fewer axonal tracts may exist within specific regions. The gray and white matter damage is accompanied by corresponding functional deficits, as shown in Figure 1 and in our previous reports [31,41].

## 4. Discussion

The present study provides novel structural evidence of sensory deficits in a recently identified SAD model of the GluN3A KO mouse. In aging/aged GluN3A KO mice at 18 months old (equivalent to ~60–70-year-old humans), cognitive deficits and severe sensory dysfunctions were validated, providing information in addition to the AD phenotype observed in young adult and middle-aged GluN3A KO mice [31,42]. Using diffusion tensor MRI, we now identified that the deficiency of the NMDAR subunit GluN3A caused widespread connectivity disruptions and regional atrophy in white and gray matter, involving the olfactory and visual systems, which were not observed in the normally aging control brain. These results highlight the crucial role of the GluN3A gene in maintaining the circuit integrity of sensory networks and projections to the cortex and hippocampus, which are essential structures for high brain functions. The observation is consistent with the proposition that chronic NMDAR hyperactivity, minor but long-lasting disruptions of Ca^2+^ homeostasis, and the resultant degenerative excitotoxicity are causal factors of sensory damage, which is a landmark deficit in AD and a significant early and late symptom of SAD.

Of note, the absence of the regulatory GluN3A does not result in a disease phenotype after birth and in young ages [31,33]. Young GluN3A KO mice of 2–3 months old display enhanced synaptic plasticity, such as increased long-term potentiation (LTP) and better learning and memory compared to WT controls [33]. This early enhancement is expected according to the physiological effect of acute/subacute hyperactivation of NMDARs [31,43]. Along with aging, GluN3A KO mice develop progressive syndromes of sensory, sensorimotor, psychological, and cognitive problems due to slowly evolving degenerative excitotoxicity [31].

In a human study of the primary visual system in 93–102-year-old subjects, amyloid deposition was not related to the dementia process [44]. In the 5xFAD mouse, a recent investigation observed decreased densities of retinal ganglion cell axon terminals and fewer thalamocortical neuron cell bodies. Further analysis showed that Aβ plaque formation played only a minor role in visual system dysfunction [26]. In the GluN3A KO mouse, the genetic factor of the GluN3A deletion/deficiency clearly mediates the pathological and functional impairments.

However, other genetic and pathologic events may contribute to or exacerbate AD progression. For example, Presenilin-1 (PSEN1) and PSEN2, the catalytic core of γ-secretase, can play a role independent of amyloid pathogenesis, which is associated with their effects on Ca^2+^ homeostasis and Aβ-independent neurodegeneration [45,46]. It is thus necessary and important to delineate how mutations of PSEN1 and/or PSEN2 alone or together with the NMDAR deficiency can affect the Ca^2+^-mediated degenerative excitotoxicity and SAD progression.

Human studies have identified that about 2–10% of populations carry mutated GluN3 gene *GRIN3* variants that can be null alleles with complete deficiency (e.g., *GRIN3A* mutates in ~2% of Han Chinese and Japanese) and *GRIN3B* variants in more than 10% of European-American populations [47,48,49]. Moreover, the genetic variation of *GRIN3A* was suggested to be a risk factor for AD [50]. Consistently, we detected reduced GluN3A expression in the cortex of human AD brains, while the GluN1 level was unchanged [31]. It appears that the percentage of *GRIN3* mutations is in close proximity to the prevalence of AD cases (1.6–6.4%) across the globe [51]. We propose that it is essential and justifiable to investigate the correlation between *GRIN3* mutation/dysfunction and human AD/ADRD. Supporting the key role of GluN3A in AD/SAD pathogenesis, we demonstrated that correcting the GluN3A deficiency by re-expression (knock-in) of this subunit in the KO brain [42] and a chronic therapy started from the preclinical stage with memantine to suppress extrasynaptic NMDARs, where GluN3A is located, effectively prevent AD progression in the GluN3A KO mouse and the classical 5xFAD mouse [31,41,52].

The visual system is increasingly recognized as a vulnerable target in AD, with both structural and functional deteriorations in AD patients [53]. Studies using advanced imaging techniques have revealed significantly reduced retinal nerve fiber layer and detected optic nerve degeneration in individuals with AD, suggesting that the disease extends beyond cognitive regions to affect sensory processing pathways [54]. Our study uncovered significant impairments in the visual system of the GluN3A KO mouse. Specifically, we observed reduced connectivity and volume in the SC, a critical component of the visual processing pathway [55], as well as decreased tract density between the SC and LGN, and LGN and V1. The difficulty of these mice in detecting the platform indicates a decline in visual acuity, which mirrors the visual disturbances often reported in AD patients [56].

In this study, we probed structural alterations in aging/aged mice of 18 months old, when the AD phenotype has well progressed. The observation is valuable for demonstrating the structural deterioration of brain regions in this SAD mouse. As the first study in the mouse model, the observation should be validated by studies of large groups, and sex differences should be delineated. Future MRI investigations at younger ages are important in understanding early changes in the sensory network. Results from young and middle-aged adults of this and other AD models will help explain the early sensory system syndrome and verify the feasibility of using non-invasive MRI as a clinical imaging marker in the preclinical stage of AD/ADRD. Furthermore, early treatments at the preclinical stage targeting these systems may ameliorate or prevent disease development [41]. This early approach is supported by observations that olfactory stimulation in animal models attenuates the development of dementia [13], and correcting eye conditions, such as cataracts, and improving vision in humans may reduce the risk of developing dementia [57].

White matter degeneration is a well-established feature of AD and has been linked to cognitive decline in both human patients and animal models [58]. A key finding of our study is the axonal impairment of the GluN3A KO mouse, as shown by whole-brain tractography. Both the number and volume of axonal tracts were significantly reduced in the GluN3A KO brain, indicating a global deterioration of the white matter integrity. Equally important, quantified analysis revealed a significant volumetric decrease in the OB and a trend of hippocampal atrophy in GluN3A KO mice compared to WT controls. This is consistent with previous studies linking vision damage and hippocampal atrophy to AD progression [59,60], which can be one of the earliest detectable structural changes in AD [61]. Critical for memory processing and formation, the hippocampus is particularly vulnerable to Ca^2+^ dysregulation. The situation can be exacerbated by the deficiency of GluN3A, a unique and critical regulatory subunit that modulates NMDAR-mediated Ca^2+^ influx [29,37,62].

In summary, this investigation highlights the pivotal role of GluN3A in maintaining neural circuit integrity and preventing neurodegeneration. The structural and functional impairments observed in GluN3A KO mice provide valuable insights into the pathogenic mechanism underlying SAD and ADRD, offering potential avenues for early diagnosis and therapeutic interventions.

## Figures and Tables

**Table 1:** Olfactory and hippocampal atrophy and alterations of connecting axonal tracts in the GluN3A KO brain.

Measurement	Region of Interest	WT	GluN3A KO	*p* Value
Regional Volume (mm^3^)	Olfactory bulb (OB)	9.30 ± 0.68	7.25 ± 1.27	0.0059 **
	Hippocampus	9.55 ± 0.44	8.83 ± 0.76	0.0733
Number of Connective tracts between OB and hippocampus	In right hemisphere	282 ± 264	207 ± 155	0.5637
	In left hemisphere	155 ± 130	76 ± 54	0.0955
	Bilateral tracts	411 ± 287	267 ± 167	0.3142

** Significantly different from normal aging controls; N = 4 brains in the WT and GluN3A KO groups, respectively.

## Data Availability

Our experimental data are available in this and other published papers; additional information can be obtained from the corresponding author.

## References

[1] Hardy J.A., Higgins G.A. (1992). Alzheimer’s disease: The amyloid cascade hypothesis. Science.

[2] Caselli R.J., Knopman D.S., Bu G. (2020). An agnostic reevaluation of the amyloid cascade hypothesis of Alzheimer’s disease pathogenesis: The role of APP homeostasis. Alzheimer’s Dement..

[3] Canter R.G., Penney J., Tsai L.H. (2016). The road to restoring neural circuits for the treatment of Alzheimer’s disease. Nature.

[4] Scheltens N.M., Galindo-Garre F., Pijnenburg Y.A., van der Vlies A.E., Smits L.L., Koene T., Teunissen C.E., Barkhof F., Wattjes M.P., Scheltens P. (2016). The identification of cognitive subtypes in Alzheimer’s disease dementia using latent class analysis. J. Neurol. Neurosurg. Psychiatry.

[5] Jack C.R., Holtzman D.M. (2013). Biomarker modeling of Alzheimer’s disease. Neuron.

[6] Ingelsson M., Fukumoto H., Newell K.L., Growdon J.H., Hedley-Whyte E.T., Frosch M.P., Albert M.S., Hyman B.T., Irizarry M.C. (2004). Early Abeta accumulation and progressive synaptic loss, gliosis, and tangle formation in AD brain. Neurology.

[7] Hong S., Baek S.H., Lai M.K.P., Arumugam T.V., Jo D.G. (2024). Aging-associated sensory decline and Alzheimer’s disease. Mol. Neurodegener..

[8] Leonenko G., Shoai M., Bellou E., Sims R., Williams J., Hardy J., Escott-Price V., Alzheimer’s Disease Neuroimaging I. (2019). Genetic risk for alzheimer disease is distinct from genetic risk for amyloid deposition. Ann. Neurol..

[9] Son G., Jahanshahi A., Yoo S.J., Boonstra J.T., Hopkins D.A., Steinbusch H.W.M., Moon C. (2021). Olfactory neuropathology in Alzheimer’s disease: A sign of ongoing neurodegeneration. BMB Rep..

[10] Son G., Steinbusch H.W.M., Lopez-Iglesias C., Moon C., Jahanshahi A. (2022). Severe histomorphological alterations in post-mortem olfactory glomeruli in Alzheimer’s disease. Brain Pathol..

[11] Rahayel S., Frasnelli J., Joubert S. (2012). The effect of Alzheimer’s disease and Parkinson’s disease on olfaction: A meta-analysis. Behav. Brain Res..

[12] Daulatzai M.A. (2015). Olfactory dysfunction: Its early temporal relationship and neural correlates in the pathogenesis of Alzheimer’s disease. J. Neural Transm..

[13] Alonso M., Lepousez G., Sebastien W., Bardy C., Gabellec M.M., Torquet N., Lledo P.M. (2012). Activation of adult-born neurons facilitates learning and memory. Nat. Neurosci..

[14] Grelat A., Benoit L., Wagner S., Moigneu C., Lledo P.M., Alonso M. (2018). Adult-born neurons boost odor-reward association. Proc. Natl. Acad. Sci. USA.

[15] Thal D.R., Rub U., Orantes M., Braak H. (2002). Phases of A beta-deposition in the human brain and its relevance for the development of AD. Neurology.

[16] Wang J., Eslinger P.J., Doty R.L., Zimmerman E.K., Grunfeld R., Sun X., Meadowcroft M.D., Connor J.R., Price J.L., Smith M.B. (2010). Olfactory deficit detected by fMRI in early Alzheimer’s disease. Brain Res..

[17] Peters J.M., Hummel T., Kratzsch T., Lotsch J., Skarke C., Frolich L. (2003). Olfactory function in mild cognitive impairment and Alzheimer’s disease: An investigation using psychophysical and electrophysiological techniques. Am. J. Psychiatry.

[18] Marin C., Vilas D., Langdon C., Alobid I., Lopez-Chacon M., Haehner A., Hummel T., Mullol J. (2018). Olfactory Dysfunction in Neurodegenerative Diseases. Curr. Allergy Asthma Rep..

[19] Woodward M.R., Amrutkar C.V., Shah H.C., Benedict R.H., Rajakrishnan S., Doody R.S., Yan L., Szigeti K. (2017). Validation of olfactory deficit as a biomarker of Alzheimer disease. Neurol. Clin. Pract..

[20] Attems J., Walker L., Jellinger K.A. (2015). Olfaction and Aging: A Mini-Review. Gerontology.

[21] Ferguson E.L., Thoma M., Buto P.T., Wang J., Glymour M.M., Hoffmann T.J., Choquet H., Andrews S.J., Yaffe K., Casaletto K. (2024). Visual Impairment, Eye Conditions, and Diagnoses of Neurodegeneration and Dementia. JAMA Netw. Open.

[22] London A., Benhar I., Schwartz M. (2013). The retina as a window to the brain-from eye research to CNS disorders. Nat. Rev. Neurol..

[23] Shafiee N., Fonov V., Dadar M., Spreng R.N., Collins D.L. (2024). Degeneration in Nucleus basalis of Meynert signals earliest stage of Alzheimer’s disease progression. Neurobiol. Aging.

[24] Nagarajan N., Assi L., Varadaraj V., Motaghi M., Sun Y., Couser E., Ehrlich J.R., Whitson H., Swenor B.K. (2022). Vision impairment and cognitive decline among older adults: A systematic review. BMJ Open.

[25] Grigorian A., McKetton L., Schneider K.A. (2016). Measuring Connectivity in the Primary Visual Pathway in Human Albinism Using Diffusion Tensor Imaging and Tractography. J. Vis. Exp..

[26] McCool S., Smith J.C., Sladek A., Fan S., Van Hook M.J. (2025). Retinal and thalamic alterations in the 5xFAD mouse model of Alzheimer’s disease. PLoS ONE.

[27] Zeppieri M., Visalli F., Musa M., Avitabile A., Giglio R., Tognetto D., Gagliano C., D’Esposito F., Cappellani F. (2025). Beyond the Eye: Glaucoma and the Brain. Brain Sci..

[28] Nam Y., Kim S., Kim J., Hoe H.S., Moon M. (2022). Mesoscopic Mapping of Visual Pathway in a Female 5XFAD Mouse Model of Alzheimer’s Disease. Cells.

[29] Yu S.P., Jiang M.Q., Shim S.S., Pourkhodadad S., Wei L. (2023). Extrasynaptic NMDA receptors in acute and chronic excitotoxicity: Implications for preventive treatments of ischemic stroke and late-onset Alzheimer’s disease. Mol. Neurodegener..

[30] Kullmann D.M., Lamsa K.P. (2007). Long-term synaptic plasticity in hippocampal interneurons. Nat. Rev. Neurosci..

[31] Zhong W., Wu A., Berglund K., Gu X., Jiang M.Q., Talati J., Zhao J., Wei L., Yu S.P. (2021). Pathogenesis of sporadic Alzheimer’s disease by deficiency of NMDA receptor subunit GluN3A. Alzheimer’s Dement..

[32] Chakroborty S., Stutzmann G.E. (2011). Early calcium dysregulation in Alzheimer’s disease: Setting the stage for synaptic dysfunction. Sci. China Life Sci..

[33] Mohamad O., Song M., Wei L., Yu S.P. (2013). Regulatory roles of the NMDA receptor GluN3A subunit in locomotion, pain perception and cognitive functions in adult mice. J. Physiol..

[34] Chen Y.Y., Chang C.J., Liang Y.W., Tseng H.Y., Li S.J., Chang C.W., Wu Y.T., Shao H.H., Chen P.C., Lai M.L. (2024). Utilizing diffusion tensor imaging as an image biomarker in exploring the therapeutic efficacy of forniceal deep brain stimulation in a mice model of Alzheimer’s disease. J. Neural Eng..

[35] Albadawi E.A. (2024). Microstructural Changes in the Corpus Callosum in Neurodegenerative Diseases. Cureus.

[36] Yu S.P. Functional and structural impairments of the olfactory and visual systems of a sporadic Alzheimer’s disease model of the GluN3A knockout mouse. Presented at the 18th Pan Pacific Symposium on Stem Cells and Cancer Research (PPSSC).

[37] Das S., Sasaki Y.F., Rothe T., Premkumar L.S., Takasu M., Crandall J.E., Dikkes P., Conner D.A., Rayudu P.V., Cheung W. (1998). Increased NMDA current and spine density in mice lacking the NMDA receptor subunit NR3A. Nature.

[38] Pinto L.H., Enroth-Cugell C. (2000). Tests of the mouse visual system. Mamm. Genome.

[39] Pitts M.W. (2018). Barnes Maze Procedure for Spatial Learning and Memory in Mice. Bio-Protocol.

[40] Salay L.D., Ishiko N., Huberman A.D. (2018). A midline thalamic circuit determines reactions to visual threat. Nature.

[41] Yu S.P., Gu X., Jiang M.Q., Sastry A., Wu L., Li Y., Wei L. (2025). Combined Preventive and Preconditioning Treatments for the Comorbidity of Alzheimer’s Disease and Ischemic Stroke in a GluN3A Knockout Mouse and a 5xFAD Mouse. Cells.

[42] Yu S.P., Jiang M., Berglund K., Wei L. (2023). Strain hypothesis and additional evidence of the GluN3A deficiency-mediated pathogenesis of Alzheimer’s disease. Alzheimer’s Dement..

[43] Yu S.P., Choi E., Jiang M.Q., Wei L. (2025). Acute and chronic excitotoxicity in ischemic stroke and late-onset Alzheimer’s disease. Neural Regen. Res..

[44] Leuba G., Saini K., Zimmermann V., Giannakopoulos P., Bouras C. (2001). Mild amyloid pathology in the primary visual system of nonagenarians and centenarians. Dement. Geriatr. Cogn. Disord..

[45] Yang G., Xu X., Xu Y. (2026). Presenilin deficiency beyond amyloid: Lessons from presenilin 1/2 conditional double-knockout mice on synaptic failure, calcium dyshomeostasis, and inflammation-driven Alzheimer’s disease. Brain Res. Bull..

[46] Parvand M., Liang J.J.H., Bozorgmehr T., Born D., Luna Cortes A., Rankin C.H. (2024). A familial Alzheimer’s disease associated mutation in presenilin-1 mediates amyloid-beta independent cell specific neurodegeneration. PLoS ONE.

[47] Tarabeux J., Kebir O., Gauthier J., Hamdan F.F., Xiong L., Piton A., Spiegelman D., Henrion E., Millet B., Team S.D. (2011). Rare mutations in N-methyl-D-aspartate glutamate receptors in autism spectrum disorders and schizophrenia. Transl. Psychiatry.

[48] Niemann S., Kanki H., Fukui Y., Takao K., Fukaya M., Hynynen M.N., Churchill M.J., Shefner J.M., Bronson R.T., Brown R.H. (2007). Genetic ablation of NMDA receptor subunit NR3B in mouse reveals motoneuronal and nonmotoneuronal phenotypes. Eur. J. Neurosci..

[49] Takata A., Iwayama Y., Fukuo Y., Ikeda M., Okochi T., Maekawa M., Toyota T., Yamada K., Hattori E., Ohnishi T. (2013). A population-specific uncommon variant in GRIN3A associated with schizophrenia. Biol. Psychiatry.

[50] Liu H.P., Lin W.Y., Liu S.H., Wang W.F., Tsai C.H., Wu B.T., Wang C.K., Tsai F.J. (2009). Genetic variation in N-methyl-D-aspartate receptor subunit NR3A but not NR3B influences susceptibility to Alzheimer’s disease. Dement. Geriatr. Cogn. Disord..

[51] Qiu C., Kivipelto M., von Strauss E. (2009). Epidemiology of Alzheimer’s disease: Occurrence, determinants, and strategies toward intervention. Dialogues Clin. Neurosci..

[52] Gu X., Yu S.P., Jiang M.Q., Zhong W., Sastry A., Wei L. (2026). Preventive Memantine Treatment at the Preclinical Stage in the Alzheimer’s Disease Model of the 5xFAD Mouse. Neurosci. Bull..

[53] Braak H., Del Tredici K. (2015). The preclinical phase of the pathological process underlying sporadic Alzheimer’s disease. Brain.

[54] Hardy J., Selkoe D.J. (2002). The amyloid hypothesis of Alzheimer’s disease: Progress and problems on the road to therapeutics. Science.

[55] Hao X., Zhang W., Jiao B., Yang Q., Zhang X., Chen R., Wang X., Xiao X., Zhu Y., Liao W. (2023). Correlation between retinal structure and brain multimodal magnetic resonance imaging in patients with Alzheimer’s disease. Front. Aging Neurosci..

[56] Zhang S., Fan Z., Ji D. (2025). Advances in eye-brain axis: Anatomy, immunity, and association with visual dysfunction. Ageing Res. Rev..

[57] Hecht I., Kanclerz P., Tuuminen R. (2023). Secondary outcomes of lens and cataract surgery: More than just “best-corrected visual acuity. Prog. Retin. Eye Res..

[58] Amlien I.K., Fjell A.M. (2014). Diffusion tensor imaging of white matter degeneration in Alzheimer’s disease and mild cognitive impairment. Neuroscience.

[59] Bathini P., Brai E., Balin B.J., Bimler L., Corry D.B., Devanand D.P., Doty R.L., Ehrlich G.D., Eimer W.A., Fulop T. (2024). Sensory Dysfunction, Microbial Infections, and Host Responses in Alzheimer’s Disease. J. Infect. Dis..

[60] Donato L., Morda D., Scimone C., Alibrandi S., D’Angelo R., Sidoti A. (2023). Bridging Retinal and Cerebral Neurodegeneration: A Focus on Crosslinks between Alzheimer-Perusini’s Disease and Retinal Dystrophies. Biomedicines.

[61] Selkoe D.J., Hardy J. (2016). The amyloid hypothesis of Alzheimer’s disease at 25 years. EMBO Mol. Med..

[62] Lee J.H., Wei Z.Z., Chen D., Gu X., Wei L., Yu S.P. (2015). A neuroprotective role of the NMDA receptor subunit GluN3A (NR3A) in ischemic stroke of the adult mouse. Am. J. Physiol.-Cell Physiol..

